# Modus Operandi of Cod-liver Oil

**Published:** 1852-02

**Authors:** 


### MODUS OPERANDI OF COD-LIVER OIL.

Dr. Carpenter, in his Principles of Physiology, speaks of cod-liver oil "as by far the most important among the recent improvements in medical practice," and thinks its curative agency may be explained upon the principle, "that the presence of fatty matter is not less essential to the formative operations, than is that of the albuminous compounds themselves·" It unites with the albumen to form the nuclei of growing cells or fibres. In a word, it is indispensable to the process of nutrition. Cod-liver oil is remarkable for improving the nutrition of tubercular subjects, as the oleaginous diet of the Icelanders is found to exert a great prophylactic influence upon this people, whose habits are such as would peculiarly favor the development of scrofula, but who are most remarkably free from any form of it. Dr. C. adds, that "the importance of oleaginous matter to the process of textural nutrition, and the probable *rationale* of the beneficial effects of cod-liver oil, were first developed by his friend, Prof. J. H. Bennett. Whatever be the rationale of its action, its efficacy is placed every day more and more beyond question.

